# Whole-Transcriptome Analysis of Gene Expression in Canine Splenic Lymphoid Hyperplasia, Complex Hyperplasia, Histiocytic Sarcoma, and Stromal Sarcoma

**DOI:** 10.3390/ani16030422

**Published:** 2026-01-29

**Authors:** Cleide Spröhnle-Barrera, Rachel Allavena, Chiara Palmieri

**Affiliations:** School of Veterinary Science, The University of Queensland, Gatton Campus, Gatton, QLD 4343, Australia; cleide-sprohnle-barrera@idexx.com (C.S.-B.);

**Keywords:** dog, spleen, RNA sequencing, nodules

## Abstract

Splenic nodules in dogs that were historically classified under the broad term “fibrohistiocytic nodules” are now recognised as distinct entities within likely a biological continuum. These include lymphoid hyperplasia extending to indolent lymphoma and complex hyperplasia to stromal sarcoma. However, the molecular mechanisms underpinning these proposed progressions remain largely unexplored, particularly at the genomic and transcriptomic levels. This study aimed to delineate and compare the transcriptomic landscapes of four distinct canine splenic nodules through differential gene expression profiling. RNA sequencing was performed on twelve formalin-fixed, paraffin-embedded (FFPE) splenic tissue samples obtained from dogs diagnosed with lymphoid hyperplasia, complex hyperplasia, histiocytic sarcoma, and stromal sarcoma, with normal canine spleen serving as a control tissue. Comparative transcriptomic analysis identified 47 differentially expressed genes (DEGs) between splenic nodules and normal spleen, including *CSRP1*, *SLC40A1*, *C1QA*, *C1QC*, *DLA-12*, *FTL*, *FXYD6*, *MPEG1*, *OAS3*, *CSF1*, and *JMJD6*. Furthermore, 39 DEGs were significantly altered among the four splenic lesion types, such as *MLC1*, *ERAS*, *MOV10L1*, *LOC102152143*, *COL4A1*, *COL4A2*, *COL12A1*, *NOTCH3*, *PLOD2*, *CPXM2*, *MRC1*, *GALNT5*, *TIMP1*, and *TFPI2*. Many of these genes have previously been implicated in tumorigenesis and metastasis in other malignancies. These findings suggest that dysregulated gene expression may contribute to the activation of stromal cells and macrophages within the spleen, facilitating malignant transformation. Overall, these findings deliver novel transcriptomic insights into canine splenic tumorigenesis that may improve diagnostic precision, inform prognostic assessment, and support the development of targeted therapeutic strategies in veterinary oncology.

## Simple Summary

Understanding diseases of the spleen in dogs is challenging because different types of splenic nodules have traditionally been grouped under a single name, despite showing very different biological behaviour. These nodules can range from benign tissue changes to aggressive cancers, making accurate diagnosis and treatment decisions difficult. The aim of this study was to improve our understanding of how these splenic nodules differ at a molecular level by examining patterns of gene activity. We compared spleen tissue from dogs with four distinct types of splenic nodules to normal spleen tissue. The results showed consistent differences in gene activity between normal spleen and nodular tissue, as well as clear differences among the four nodule types. Many of the affected genes are known to be involved in cancer development and progression in other diseases. These findings suggest that altered gene activity may contribute to the transition from non-cancerous splenic changes to malignant disease. This study provides new insights into the biological mechanisms underlying splenic tumours in dogs and may support the development of improved diagnostic methods and more targeted treatments, benefiting canine health and veterinary care.

## 1. Introduction

In the canine spleen, nodular masses are a common finding and may represent benign lesions, malignant neoplasms, or non-neoplastic proliferations. According to recent retrospective analyses, the most common malignant diagnosis remains haemangiosarcoma (HSA), with non-neoplastic hyperplasia dominating the benign lesions [1]. Because of its relative frequency and aggressive behaviour, HSA remains the most extensively studied splenic tumour in dogs [2]. However, other entities such as hyperplastic nodules, histiocytic sarcoma, and stromal sarcoma are also frequently diagnosed in splenectomised dogs [3]. These non-vascular tumours were previously categorised as “splenic fibrohistiocytic nodules” (SFHN), denoting a heterogeneous group of malignant, and non-malignant growths that share specific morphological features such as a distinct lymphoid population and/or fibrohistiocytic elements [4]. Differentiating splenic nodules is challenging because their morphological features often overlap, making these conditions difficult to distinguish. In a study of 31 dogs initially diagnosed with SFHN, histologic and immunohistochemical re-evaluation identified 13 nodular hyperplasias, 4 lymphomas, 8 stromal sarcomas, and 6 histiocytic sarcomas, demonstrating that previously classified fibrohistiocytic nodules were often misdiagnosed or misclassified [5]. A study of 109 splenic cases demonstrated that fibrohistiocytic nodules form a continuum from lymphoid hyperplasia with spindle and histiocytic cells to overt fibrohistiocytic neoplasms, emphasising the need for accurate histopathologic evaluation [6]. Corroborating this concept, splenic nodular lesions retaining the overall architecture and morphological features consistent with complex nodular hyperplasias (CNHs) but containing a focal (<50%) region of the stromal component similar to undifferentiated stromal sarcomas are now classified as sarcomas arising from CNH [7]. Thus, the term “fibrohistiocytic nodule” is now discouraged in favour of specific entities like lymphoid nodular hyperplasia (LNH), complex nodular hyperplasia, undifferentiated splenic stromal sarcoma (excluding other splenic stromal sarcomas with a well-defined phenotype, such as fibrosarcoma, myxosarcoma, leiomyosarcoma, liposarcoma) whether arising from complex nodular hyperplasia or not, and histiocytic sarcoma [8,9].

These studies show that conventional histology often misdiagnoses splenic nodules, making advanced techniques like immunohistochemistry essential. Immunohistochemistry, used alongside H&E staining, helps accurately differentiate splenic stromal sarcoma from other malignancies like histiocytic sarcoma or lymphoma, which is critical for prognosis and treatment decisions [8]. For instance, studies proposed a combination of IHC markers such as CD3 (T-lymphocytes), BLA.36 (B-lymphocyte marker), CD18 (histiocytic marker), and lysozyme (histiocytes) to identify specific cellular lineages and differentiate between lymphoid, histiocytic, and mesenchymal tumours [5,6,8]. Although advances in immunohistochemistry have improved diagnostic accuracy on potential pre-operative needle core biopsies, diagnoses are often made post surgically. Currently, ultrasound-guided fine-needle aspiration (FNA) of the spleen is one of the most commonly performed diagnostic procedures to rule in malignancy; however, agreement between cytopathologic and histopathologic diagnoses is only 61.3% [10]. An initial study evaluating the addition of splenic needle core biopsy (NCB) during splenic sampling demonstrated improved discrimination between neoplastic and non-neoplastic lesions and enhanced lesion subclassification [11]. Nevertheless, the diagnostic performance of these techniques remains limited: both FNA and NCB demonstrated a sensitivity of 53–71%, while specificity was 75–88% [12]. The development of cancer-specific biomarkers and a deeper understanding of neoplastic growth and progression could support current diagnostic procedures, enhance diagnostic resolution, refine prognostic stratification, and enable earlier or non-invasive detection [13]. This is particularly relevant considering that surgical removal of the spleen (splenectomy), though often curative or diagnostic, carries inherent risks, including peri-operative haemorrhage, immune dysregulation, and altered haematopoiesis [14].

Understanding the biology of the tumour microenvironment is essential for integrating diagnostic innovations, such as cancer biomarker identification, with effective therapeutic strategies. While gene expression profiling in canine tumours has primarily focused on osteosarcoma [15], melanoma [16], mammary [17], prostate [18], and bladder carcinomas [19], few studies have addressed splenic nodular lesions. A recent miRNA profiling study identified 22 differentially expressed miRNAs between canine splenic HSA, nodular hyperplasia, and normal spleen, including miRNAs involved in angiogenesis (miR-26a, miR-126, miR-150) and others linked to cancer pathways (miR-22, miR-452) [20]. This study demonstrates that RNA-seq and other high-throughput sequencing methods offer the prospect of gaining deeper insights into the pathogenesis and progression of splenic tumours. Another recent study on the tumour immune microenvironment in canine splenic HSA (n = 56) reported that specific tumour-infiltrating lymphocyte (TIL) subsets (for example FoxP3+ regulatory T-cells, CD20+ B-cells) correlated with tumour-related death, metastasis, and survival time [21]. Together, these studies highlight the need for more comprehensive biological characterisation of canine splenic nodules to enable early detection, accurate patient classification, and the development of targeted, immune-informed therapies tailored to the tumour microenvironment.

Despite advances in histopathological and immunophenotypic characterization, the molecular underpinnings of canine splenic nodular lesions remain poorly defined. In particular, the potential biological continuum linking hyperplastic, inflammatory, and neoplastic processes within the spleen has not been explored at the transcriptomic level. Therefore, the present study aimed to characterize and compare the gene expression profiles of distinct canine splenic nodules including lymphoid hyperplasia, complex hyperplasia, histiocytic sarcoma, and stromal sarcoma using RNA sequencing. By integrating comparative transcriptomic analyses with current histopathological classifications, this work seeks to identify key molecular pathways and candidate biomarkers involved in splenic tumorigenesis, thereby providing new insights into the pathogenesis, progression, and potential diagnostic stratification of these clinically significant lesions.

## 2. Materials and Methods

### 2.1. Case Identification and Inclusion Criteria

Canine splenic nodular lesions were retrospectively retrieved from pathology archives of the Veterinary Laboratory Service of the University of Queensland, Australia, between 1994 and 2019. Patients diagnosed with splenic lymphoid nodular hyperplasia, complex nodular hyperplasia, histiocytic sarcoma, and stromal sarcoma were selected. Haematoxylin and eosin (H&E)-stained slides were reviewed by a Board-Certified Veterinary Pathologist. Three representative cases per lesion type (twelve total) were selected based on tissue quality, availability of >0.5 cm^2^ of formalin-fixed paraffin-embedded (FFPE) tissue, minimal necrosis, and good preservation. Animal ethics approval for usage of splenic tissue collected from animals already undergoing diagnostic procedures was granted by the University of Queensland Animal Ethics Committee under certificate numbers 2020/AE000076ANRFA and 2020/AE000074ANRFA.

### 2.2. Selection for Histopathology and Immunohistochemistry

Immunophenotyping was performed on the selected cases using CD3 (T-cells), CD79a (B-cells), MAC-387 and IBA-1 (macrophages/histiocytes), and vimentin (mesenchymal cells). FFPE sections (5 µm) were mounted on poly-L-lysine-coated slides, deparaffinized, rehydrated, and subjected to heat-induced antigen retrieval. Endogenous peroxidase was blocked, followed by incubation with primary antibodies and detection using the EnVision™ FLEX/HRP system (Agilent Technologies Australia, Mulgrave, Victoria, Australia) with DAB chromogen and Mayer’s haematoxylin counterstaining (Table 1). Negative controls included sections incubated with antibody diluent only. FFPE spleen tissue from a healthy dog served as a normal tissue control. All antibodies were validated for canine cross-reactivity as per previously published studies.

### 2.3. Selection for RNA-Seq Analysis

Three FFPE blocks were selected from each diagnostic group: lymphoid hyperplasia, complex hyperplasia, histiocytic sarcoma, and stromal sarcoma were selected, along with a healthy spleen control. Seven 10 µm thick sections were cut from each block and submitted to BGI Genomics (Hong Kong) for RNA extraction and sequencing.

### 2.4. RNA Sequencing and Bioinformatic Workflow

Transcriptome libraries were prepared using the VAZYME Ribo-off rRNA Depletion Kit (Human/Mouse/Rat, N406-02) (Vazyme Biotech Co., Ltd., Nanjing, Jiangsu, China) and sequenced on the DNBSEQ G-400 platform with 100-bp paired-end reads. RNA quality and quantity were confirmed prior to DNase treatment. The corresponding sample quality control protocol included checking the amount of tissue available (>1 gr). Fragmented RNA was reverse-transcribed to generate first- and second-strand cDNA, which was subsequently fragmented, end-repaired, adenylated, and ligated to sequencing adaptors. Single-stranded circular DNA molecules were amplified via rolling circle amplification to produce DNA nanoballs (DNBs), which were loaded onto patterned nanoarrays and sequenced using combinatorial Probe-Anchor Synthesis (cPAS) (BGI genomics, Yantian District Shenzhen, Guangdong Province, China).

Bioinformatic analyses were performed through the Queensland Cyber Infrastructure Foundation (QCIF) using the Galaxy 22.05 platform. Quality control of raw reads was conducted with FastQC, (version 0.72 + galaxy 1) followed by adapter and quality trimming using FASTQ Quality Trimmer (Galaxy version 1.1.5). Reads were aligned to the *Canis familiaris* reference genome using HISAT2 (Galaxy Version 2.2.1+galaxy1), and gene expression counts were normalised prior to statistical testing.

Differential expression analysis was performed using the limma package (Galaxy Version 3.58.1+galaxy0). Genes were considered differentially expressed (DEGs) if they met the criteria of an adjusted *p*-value < 0.01 and |log_2_ fold change| > 1 [22].

## 3. Results

### 3.1. Histopathology and Immunohistochemical Examination

Tumours were classified into four groups: lymphoid hyperplasia (LH), complex hyperplasia (CH), histiocytic sarcoma (HS), and stromal sarcoma (SS). This was based on histology and immunohistochemistry patterns. Histologically, LH featured enlarged follicles with distinct germinal centres, abundant marginal zone cells, and scattered spindle cells, macrophages, and occasional granulocytes, with low mitotic activity (0–2/2.37 mm^2^). Immunohistochemically, follicles were strongly CD79a-positive (B-cells), extrafollicular areas contained numerous CD3-positive T-cells, MAC387-positive macrophages were scattered in PALs, and vimentin-positive stromal cells were widespread. The complex hyperplasia cases showed lymphoid follicles comprising 60–70% of the lesion with few mitoses, accompanied by 30–40% fibrohistiocytic tissue containing spindle and larger histiocytic cells. Follicles showed weak to moderately strong CD79a staining, while extrafollicular areas had strong CD3 positivity and increased numbers of MAC387- and vimentin-positive cells in parafollicular regions. Histiocytic sarcoma formed poorly defined nodules that effaced follicles and invaded red pulp, composed of pleomorphic round cells with occasional multinucleated forms and a high mitotic rate (>10/2.37 mm^2^). Tumour cells showed strong MAC387 and IBA-1 positivity and moderate vimentin staining, with scattered CD3 (T-cell) and CD79a (B-cell)-positive lymphocytes within nodules and more in PALs and red pulp. The stromal sarcoma cases showed highly cellular, unencapsulated spindle-cell nodules with marked pleomorphism, high mitotic rates (>10/2.37 mm^2^) with frequent extramedullary haematopoiesis, necrosis, and haemorrhage. Tumour cells were strongly vimentin-positive cells, with few scattered CD3 T-cell, CD79a B-cell, and mildly increased numbers of MAC387-positive cells.

### 3.2. RNA-Seq: Identification of Differentially Expressed Genes Between Nodular Lesions and Canine Normal Spleen

A total of 47 genes were differentially expressed between normal spleen and splenic lesions, comprising 44 downregulated and 3 upregulated transcripts (fold change ≥ 2).

### 3.3. Lymphoid Hyperplasia Versus Normal Spleen

**Figure 1:**
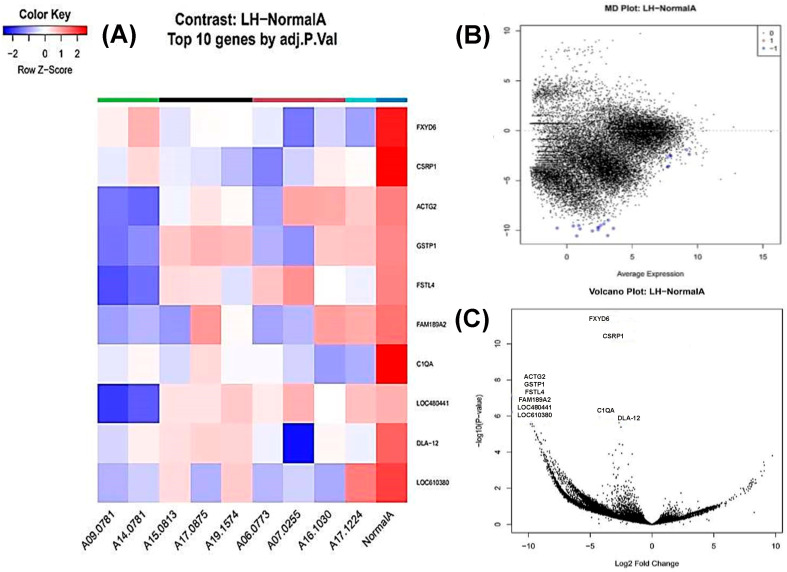
Transcriptomic profiling of lymphoid nodular hyperplasia of the canine spleen. (**A**) A heat map showing the top 10 statistically significant differentially expressed genes (DEGs). An FDR value of 0.01 was used as the significance threshold. Rows correspond to individual genes and columns to individual samples. Cell colours indicate relative gene expression levels after normalization, with red representing higher expression and blue lower expression. (**B**) An MD plot of genes differentially expressed between lymphoid hyperplasia (LH) and normal spleen. The x-axis represents the average log counts per million (log-CPM), while the y-axis represents the log2 fold change (logFC). Statistically significant genes are highlighted in blue; 21 genes are significantly downregulated in LH. (**C**) A volcano plot of differentially expressed genes (DEGs) between lymphoid hyperplasia (LH) compared to normal spleen. The x-axis represents the log2 fold change and the y-axis represents the −log10-adjusted *p* value. Genes with a fold change ≥ 2 and statistical significance are labelled using standardized gene symbols.

Twenty-one genes were differentially expressed, all showing downregulation in lymphoid hyperplasia (Figure 1). Among the top 10 DEGs, the most downregulated were *ACTG2* (actin gamma 2, smooth muscle), *GSTP1* (glutathione S-transferase pi 1), *FSTL4* (follistatin-like 4), *FAM189A2*, and *LOC610380* (corticotropin-releasing factor receptor 1) (Figure 1A–C).

### 3.4. Complex Hyperplasia vs. Normal Spleen

**Figure 2:**
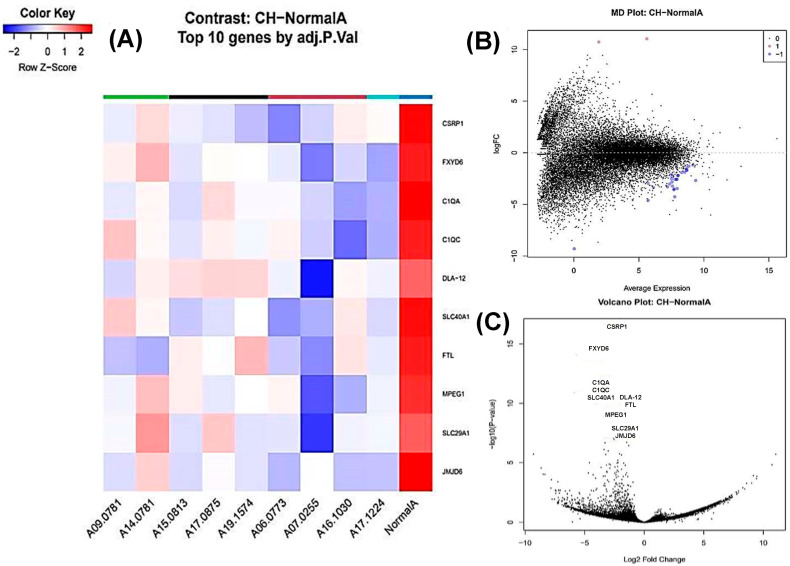
Transcriptomic profiling of complex nodular hyperplasia of the canine spleen. (**A**) A heat map showing the top 10 statistically significant differentially expressed genes (DEGs) in complex hyperplasia (CH) compared to normal spleen. An FDR value of 0.01 was used as the significance threshold. Rows correspond to individual genes and columns to individual samples. Cell colours indicate relative gene expression levels after normalization, with red representing higher expression and blue lower expression. (**B**) An MD plot of genes differentially expressed between complex hyperplasia (CH) and normal spleen. The x-axis represents the average log counts per million (log-CPM), while the y-axis represents the log2 fold change (logFC). There are 28 downregulated genes (blue) and 2 upregulated genes (red), both statistically significant. (**C**) A volcano plot of differentially expressed genes (DEGs) between complex hyperplasia (CH) compared to normal spleen. The x-axis represents the log2 fold change and the y-axis represents the −log10-adjusted *p* value. Genes with a fold change ≥ 2 and statistical significance are labelled using standardized gene symbols.

Thirty genes were differentially expressed, with twenty-eight downregulated and two upregulated (Figure 2). The top downregulated genes included *FXYD6* (FXYD domain-containing ion transport regulator 6), *C1QC* and *C1QA* (complement components), and *SLC40A1* (solute carrier family 40 member 1) (Figure 2A–C).

### 3.5. Histiocytic Sarcoma vs. Normal Spleen

**Figure 3:**
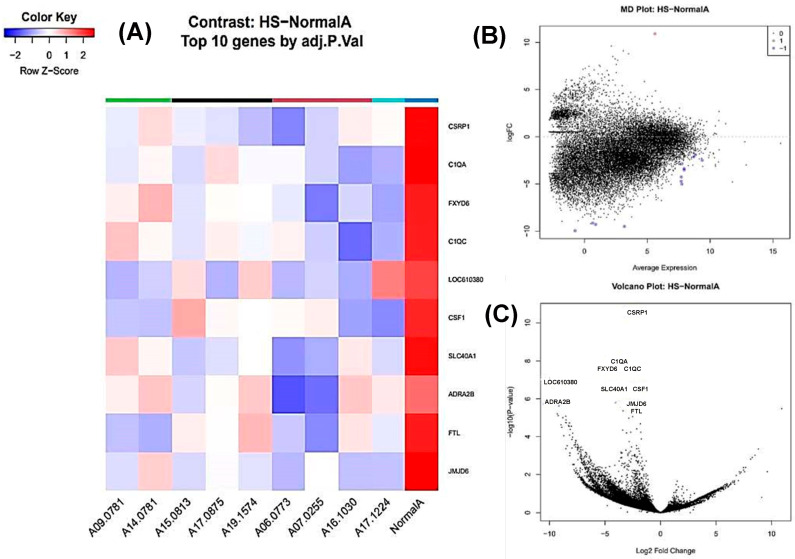
Transcriptomic profiling of histiocytic sarcoma of the canine spleen. (**A**) A h eat map showing the top 10 statistically significant differentially expressed genes (DEGs) in histiocytic sarcoma (HS) compared to normal spleen. An FDR value of 0.01 was used as the significance threshold. Rows correspond to individual genes and columns to individual samples. Cell colours indicate relative gene expression levels after normalization, with red representing higher expression and blue lower expression. (**B**) An MD plot of genes differentially expressed between histiocytic sarcoma (HS) and normal spleen. The x-axis represents the average log counts per million (log-CPM), while the y-axis represents the log2 fold change (logFC). There are 13 downregulated genes (blue) and 1 upregulated gene (red), both statistically significant. (**C**) A volcano plot of differentially expressed genes (DEGs) between histiocytic sarcoma (HS) compared to normal spleen. The x-axis represents the log2 fold change and the y-axis represents the −log10-adjusted *p* value. Genes with a fold change ≥ 2 and statistical significance are labelled using standardized gene symbols.

Fourteen genes were differentially expressed, with thirteen downregulated and one upregulated (Figure 3). Among the top DEGs, the most downregulated were *LOC610380*, *ADRA2B* (adrenoceptor alpha 2B), *FXYD6*, *C1QC*, *C1QA*, and *SLC40A1* (Figure 3A–C).

### 3.6. Stromal Sarcoma vs. Normal Spleen

**Figure 4:**
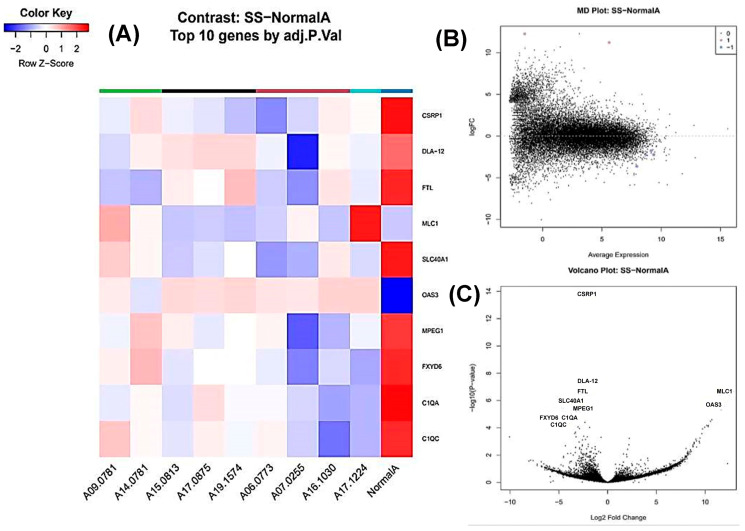
Transcriptomic profiling of splenic stromal sarcoma of the canine spleen. (**A**) A heat map showing the top 10 statistically significant differentially expressed genes (DEGs) in stromal sarcoma (SS) compared to normal spleen. An FDR value of 0.01 was used as the significance threshold. Rows correspond to individual genes and columns to individual samples. Cell colours indicate relative gene expression levels after normalization, with red representing higher expression and blue lower expression. (**B**) An MD plot of genes differentially expressed between stromal sarcoma (SS) and normal spleen. The x-axis represents the average log counts per million (log-CPM), while the y-the axis represents log2 fold change (logFC). There are 4 downregulated genes (blue) and 3 upregulated genes (red), both statistically significant. (**C**) A volcano plot of differentially expressed genes (DEGs) between stromal sarcoma (SS) compared to normal spleen. The x-axis represents the log2 fold change and the y-axis represents the −log10-adjusted *p* value. Genes with a fold change ≥ 2 and statistical significance are labelled using standardized gene symbols.

Six genes were differentially expressed, with four downregulated and two upregulated (Figure 4). The top DEGs included *SLC40A1*, *OAS3* (2’-5’-oligoadenylate synthetase 3), and *MLC1* (modulator of VRAC current 1) (Figure 4A–C).

### 3.7. Commonly Dysregulated Genes Across Lesions

Two genes, *CSRP1* (cysteine and glycine-rich protein 1) and *SLC40A1*, were consistently downregulated across all four types of splenic nodules. *C1QA*, *C1QC*, *FXYD6*, and *MPEG1* were downregulated in lymphoid hyperplasia, complex hyperplasia, and histiocytic sarcoma. *DLA-12* (MHC class I) and *FTL* (ferritin light chain) were downregulated, while *OAS3* was upregulated in lymphoid hyperplasia, complex hyperplasia, and stromal sarcoma. *CSF1* (colony-stimulating factor 1) and *JMJD6* (jumonji domain-containing 6) were similarly dysregulated in histiocytic sarcoma and complex hyperplasia. *MLC1* upregulation was unique to stromal sarcoma.

### 3.8. RNA-Seq: Differentially Expressed Genes Between Splenic Nodular Lesions

A total of 39 genes were differentially expressed among the splenic lesion types, with the majority upregulated and only one gene downregulated (fold change ≥ 2).

### 3.9. Complex Hyperplasia vs. Lymphoid Hyperplasia

**Figure 5:**
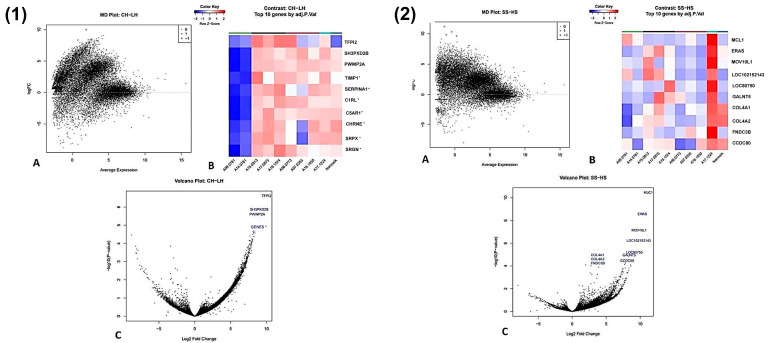
Transcriptomic comparison between lymphoid hyperplasia (LH) and complex hyperplasia (CH) (**1A**–**1C**) and stromal sarcoma (SS) and histiocytic sarcoma (HS) (**2A**–**2C**). (**1**) Lymphoid hyperplasia vs. complex hyperplasia. (**A**) An MD plot of genes with differential expression between lymphoid hyperplasia (LH) and complex hyperplasia (CH). The x-axis represents the average log counts per million (log-CPM), and the y-axis represents the log2 fold change (logFC). One gene is significantly upregulated in LH (shown in red) at the selected FDR threshold. (**B**) A heat map of statistically significant differentially expressed genes between lymphoid hyperplasia (LH) and complex hyperplasia (CH) (FDR-adjusted *p* ≤ 0.01). Rows correspond to individual genes and columns to individual samples. Colours indicate normalized relative expression levels, with red representing higher expression and blue lower expression. (**C**) A volcano plot of differentially expressed genes (DEGs) between lymphoid hyperplasia (LH) and complex hyperplasia (CH). The x-axis shows the log2 fold change and the y-axis shows the −log10-adjusted *p* value. Genes with a fold change ≥ 2 are labelled using standardized acronyms. (**2**) Stromal sarcoma vs. histiocytic sarcoma. (**A**) An MD plot showing genes with differential expression between histiocytic sarcoma and stromal sarcoma. The x-axis represents the average log counts per million (log-CPM), and the y-axis represents the log2 fold change (logFC). There are four upregulated genes (red) that are statistically significant. (**B**) A heat map of statistically significant differentially expressed genes between histiocytic sarcoma and stromal sarcoma (FDR-adjusted *p* ≤ 0.01). Rows correspond to individual genes and columns to individual samples. Colours indicate normalized relative expression levels, with red representing higher expression and blue lower expression. (**C**) A volcano plot of differentially expressed genes (DEGs) between histiocytic sarcoma and stromal sarcoma. Genes with a fold change ≥ 2 are labelled using standardized acronyms. Genes * = all the genes labelled with a * in the heat map (**B**).

Analysis identified a single gene, *TFPI2* (tissue factor pathway inhibitor 2), as significantly upregulated in complex hyperplasia compared to lymphoid hyperplasia (logFC = 10.01; adjusted *p* = 0.005) (Figure 5(1A)). Nine additional genes (*SH3PXD2B*, *PWWP2A*, *TIMP1*, *SERPINA1*, *C1RL*, *C5AR1*, *CHRNE*, *SRPX*, and *SRGN*) displayed logFC > ±2, indicating pronounced upregulation in complex hyperplasia, although these changes were not statistically significant (*p* < 0.05) (Figure 5(1B,1C)).

### 3.10. Stromal Sarcoma vs. Histiocytic Sarcoma

Four genes were significantly upregulated in stromal sarcoma relative to histiocytic sarcoma (*MLC1*, *ERAS*, *MOV10L1*, and *LOC102152143*), with five additional genes showing consistent upregulation but not reaching statistical significance (*p* < 0.05) (Figure 5(2A–2C)).

### 3.11. Histiocytic Sarcoma vs. Lymphoid/Complex Hyperplasia

**Figure 6:**
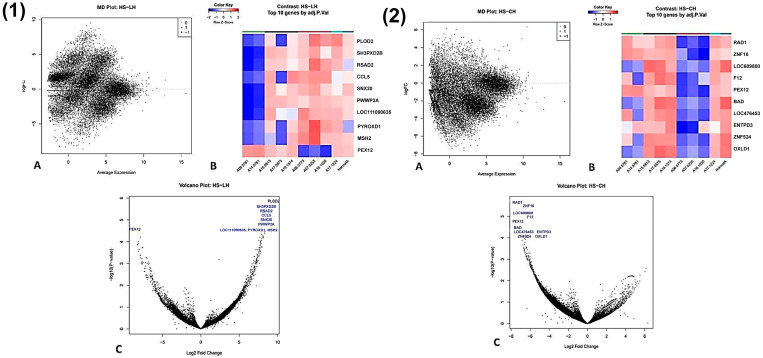
Transcriptomic comparison between lymphoid hyperplasia (LH) and histiocytic sarcoma (HS) (**1A**–**1C**) and complex hyperplasia (CH) and histiocytic sarcoma (HS) (**2A**–**2C**) and in the canine spleen. (**1**) Lymphoid hyperplasia vs. histiocytic sarcoma. (**A**) An MD plot showing genes with differential expression between lymphoid hyperplasia (LH) and histiocytic sarcoma (HS). The x-axis represents the average log counts per million (log-CPM), and the y-axis represents the log2 fold change (logFC). No genes reached statistical significance at the selected false discovery rate (FDR) threshold. (**B**) A heat map of statistically significant differentially expressed genes between lymphoid hyperplasia and histiocytic sarcoma (FDR-adjusted *p* ≤ 0.01). Rows correspond to individual genes and columns to individual samples. Colours indicate normalized relative expression levels, with red representing higher expression and blue lower expression. (**C**) A volcano plot of differentially expressed genes (DEGs) between lymphoid hyperplasia and histiocytic sarcoma. Genes with a fold change ≥ 2 are labelled using standardized acronyms. (**2**) Complex hyperplasia vs. histiocytic sarcoma. (**A**) An MD plot showing genes with differential expression between complex hyperplasia (CH) and histiocytic sarcoma (HS). The x-axis represents the average log counts per million (log-CPM), and the y-axis represents the log2 fold change (logFC). No genes reached statistical significance at the selected false discovery rate (FDR) threshold. (**B**) A heat map of statistically significant differentially expressed genes between complex hyperplasia and histiocytic sarcoma (FDR-adjusted *p* ≤ 0.01). Rows correspond to individual genes and columns to individual samples. Colours indicate normalized relative expression levels, with red representing higher expression and blue lower expression. (**C**) A volcano plot of differentially expressed genes (DEGs) between complex hyperplasia and histiocytic sarcoma. Genes with a fold change ≥ 2 are labelled using standardized acronyms.

No genes reached statistical significance (*p* < 0.01) when comparing histiocytic sarcoma to either lymphoid or complex hyperplasia. However, histiocytic sarcoma exhibited upregulation of 11 genes (*PLOD2*, *SH3PXD2B*, *RSAD2*, *CCL5*, *SNX20*, *PWWP2A*, *LOC111090635*, *PYROXD1*, *MSH2*, *LOC111097444*, and *LOC102157122*) and downregulation of 1 gene (*PEX12*) relative to lymphoid hyperplasia (*p* < 0.05) (Figure 6(1A–1C)). Compared to complex hyperplasia, histiocytic sarcoma showed downregulation of two genes (*RAD1* and *ZNF16*) without reaching statistical significance (*p* < 0.05) (Figure 6(2A–2C)).

### 3.12. Stromal Sarcoma vs. Lymphoid and Complex Hyperplasia

**Figure 7:**
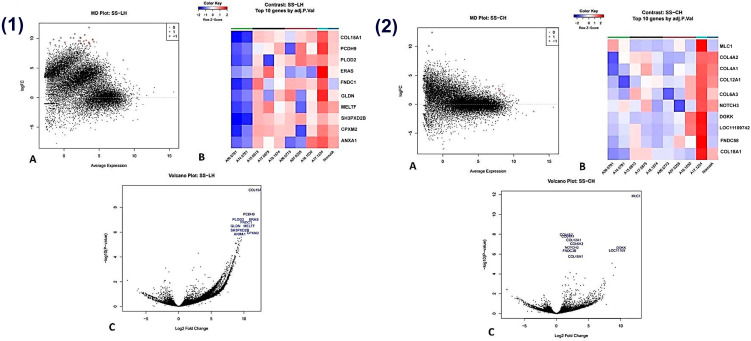
Transcriptomic comparison between stromal sarcoma (SS) and lymphoid hyperplasia (LH) (**1A**–**1C**) and stromal sarcoma (SS) and complex hyperplasia (CH) (**2A**–**2C**) in the canine spleen. (**1**) Lymphoid hyperplasia vs. stromal sarcoma. (**A**) An MD plot of genes with differential expression between lymphoid hyperplasia (LH) and stromal sarcoma (SS). The x-axis represents the average log counts per million (log-CPM), and the y-axis represents the log2 fold change (logFC). There are 28 upregulated genes (red) that are statistically significant. (**B**) A heat map of statistically significant differentially expressed genes between lymphoid hyperplasia and stromal sarcoma (FDR-adjusted *p* ≤ 0.01). Rows correspond to individual genes and columns to individual samples. Colours indicate normalized relative expression levels, with red representing higher expression and blue lower expression. (**C**) A volcano plot of differentially expressed genes (DEGs) between lymphoid hyperplasia and stromal sarcoma. The x-axis shows the log2 fold change and the y-axis shows the −log10-adjusted *p* value. Genes with a fold change ≥ 2 are labelled using standardized acronyms.(**2**) Stromal sarcoma vs. complex hyperplasia. (**A**) An MD plot of genes with differential expression between complex hyperplasia and stromal sarcoma. The x-axis represents the average log counts per million (log-CPM), and the y-axis represents the log2 fold change (logFC). There are 10 upregulated genes (red) that are statistically significant. (**B**) A heat map of statistically significant differentially expressed genes between complex hyperplasia and stromal sarcoma. (FDR-adjusted *p* ≤ 0.01). Rows correspond to individual genes and columns to individual samples. Colours indicate normalized relative expression levels, with red representing higher expression and blue lower expression. (**C**) A volcano plot of differentially expressed genes (DEGs) between complex hyperplasia and stromal sarcoma. The x-axis shows the log2 fold change and the y-axis shows the −log10-adjusted *p* value. Genes with a fold change ≥ 2 are labelled using standardized acronyms.

When compared to lymphoid hyperplasia, stromal sarcoma displayed significant upregulation of 28 genes (*p* < 0.01). The top 10 upregulated genes included *COL15A1*, *PCDH9*, *PLOD2*, *FNDC1*, *GLDN*, *ERAS*, *MELTF*, *ANXA1*, *SH3PXD2B*, and *CPXM2* (Figure 7(1A–1C)).

Compared to complex hyperplasia, stromal sarcoma showed upregulation of 10 genes (*p* < 0.01), including *MLC1*, *COL4A2*, *COL4A1*, *COL12A1*, *COL6A3*, *NOTCH3*, *DGKK*, *LOC111097427*, *FNDC3B*, and *COL18A1* (Figure 7(1A–1C)).

## 4. Discussion

Our transcriptomic analysis revealed extensive molecular heterogeneity among spontaneous canine splenic nodular lesions, reinforcing their relevance as a comparative model for investigating the molecular basis of splenic pathology. Beyond simple lesion classification, these findings suggest that splenic nodules occupy a biological continuum in which progressive transcriptional reprogramming accompanies histological complexity. Our findings highlight several differentially expressed genes (DEGs) that represent potential candidates of molecular pathways regulating the development, growth and progression of the nodular lesions under investigation (lymphoid hyperplasia, complex hyperplasia, histiocytic sarcoma, and stromal sarcoma). A summary of the significantly expressed genes among splenic nodular lesions is shown in Table 2. 

Notably, across all lesion types—both hyperplastic and neoplastic—we observed consistent downregulation of the genes *CSRP1* and *SLC40A1* compared to normal spleen. Rather than distinguishing benign from malignant lesions, these shared changes may reflect early molecular events that precede overt neoplastic transformation. Nevertheless, their persistence across lesion categories suggests that specific transcriptional changes may create a permissive background upon which additional oncogenic events accumulate. *CSRP1* (cysteine-rich protein 1), a gene involved in cytoskeletal organisation, cell growth and differentiation [23], has been proposed as tumour suppressor in human cancers, with its downregulation associated with metastatic behaviour in several tumour types, including prostate cancer [24]. In the present study, consistent CSRP1 suppression may reflect early loss of differentiation control within splenic stromal and immune compartments. It is tempting to interpret this pattern as suggestive of a stromal continuum, in which progressive cytoskeletal and polarity deregulation facilitates the transition toward proliferative or less differentiated cellular states. Similarly, downregulation of the solute carrier family 40 member 1 (*SLC40A1*)—highly expressed in macrophages and reticuloendothelial cells [25]—was a consistent event in the hyperplastic and neoplastic lesions under investigation. *SLC40A1* encodes ferroportin-1, the only known cellular iron exporter in vertebrates, and plays a crucial role in systemic and cellular iron homeostasis [26]. Thus, *SLC40A1* downregulation or functional inhibition in tumour cells or tumour-associated cells leads to intracellular iron accumulation, which may support tumour growth via increased oxidative stress regulation, DNA synthesis, or evasion of ferroptosis [26]. This dysregulated iron metabolism might actually precede neoplastic transformation, allowing for the formation of a tumour-permissive microenvironment influencing macrophage functional states. Therefore, iron homeostasis and iron metabolism pathways may represent a potentially important axis in canine splenic pathology.

Among the splenic lesion types, stromal sarcoma exhibited the highest greater degree of transcriptional divergence from normal spleen, consistent with its aggressive histological and clinical behaviour. The most significantly upregulated gene was *MLC1* (modulator of VRAC current 1), implicated in membrane transport, cell–cell interactions, and cell polarity, particularly within CNS astrocytes [27]. This transcriptional shift aligns with the histologically invasive phenotype of spindle-cell sarcomas, in which altered polarity and migratory capacity are prominent features.

This interpretation is further supported by the concurrent upregulation of *ERAS* (ES cell expressed Ras) and *NOTCH3*, implicating activation of PI3K/Akt and NOTCH signalling pathways [28]. Rather than representing isolated oncogenic events, these changes suggest coordinated stromal activation and mesenchymal transition, potentially driving fibroblast proliferation, extracellular matrix remodelling, and tumour expansion. Whether these pathways initiate sarcomagenesis or are amplified during progression remains uncertain, but their alignment with histological invasiveness supports a functional role in lesion evolution.

When comparing complex hyperplasia to stromal sarcoma, there was marked upregulation of extracellular matrix (ECM) remodelling genes *COL4A1*, *COL4A2*, and *COL12A1*. Increased expression of these genes may correspond to the invasive properties of sarcoma cells seen histologically by engaging integrin-mediated activation of downstream effectors (Src/AKT, ERK1/2, STAT3), with increased MMP-9 activity and β-catenin signalling [29,30].

Further evidence of ECM-driven progression is provided by upregulation of the enzyme *PLOD2* (lysyl hydroxylase 2) in stromal sarcoma relative to lymphoid hyperplasia. PLOD2 catalyses collagen cross-linking and fibril stabilisation, which is a key step in ECM maturation and stiffness. In human cancers, *PLOD2* overexpression correlates with increased invasion, metastasis, and poorer outcomes [31]. Its increased expression in more aggressive splenic lesions may therefore reflect adaptation of the microenvironment toward a mechanically permissive and pro-invasive state. Additional genes upregulated during the transition from lymphoid hyperplasia to stromal sarcoma, including *CPXM2* and *GALNT5*, further support progressive molecular adaptation. CPXM2 has been associated with stemness, proliferation, and migration via EMT modulation in human soft tissue sarcomas [32]. GALNT5 (polypeptide N-acetyl-galactosaminyltransferase 5) has been shown to interact with HSP90, with stabilisation of oncogenic proteins [33]. These findings raise the possibility that neoplastic stromal lesions acquire molecular features associated with adaptability and progression.

In contrast, the comparison between lymphoid hyperplasia and complex hyperplasia revealed a more limited transcriptional shift, with *TFPI2* emerging as the sole upregulated gene. TFPI2 (tissue factor pathway inhibitor 2) functions as an inhibitor of extracellular proteases involved in ECM remodelling [34] and has been linked to reduced invasion and metastasis in human cancers [35]. Its increased expression in complex hyperplasia may represent a transient protective or reactive response to increasing stromal complexity, potentially mediated by macrophages or stromal cells. Whether this response delays progression or is eventually overridden during malignant transformation remains unknown. Immune modulation emerged as a recurring theme across both hyperplastic and neoplastic lesions. Shared downregulation of *DLA-12* (dog leukocyte antigen class I, major histocompatibility complex region) suggests reduced antigen presentation and impaired immune surveillance, echoing mechanisms described in canine transmissible venereal tumour and human malignancies [36]. This transcriptional change may correspond to histological alterations in immune cell distribution and function within affected splenic regions, although targeted spatial analyses will be required to confirm this relationship.

Alterations in iron storage and immune signalling further support immune microenvironment remodelling. Downregulation of *FTL* (ferritin light chain) may reduce the ability of tumour cells to avoid oxidative stress, potentially influencing cell survival and immune interactions.

Conversely, upregulation of *OAS3* (2’-5’-oligoadenylate synthetase 3) in complex hyperplasia, histiocytic sarcoma, and stromal sarcoma suggests altered interferon-mediated antiproliferative responses, regulation of apoptosis, and immune signalling [36,37]. Recent human cancer [38] studies associate high OAS3 expression with poor outcomes, immune infiltration, and tumour progression [39], raising the possibility that interferon pathway activation reflects a dysregulated or ineffective anti-tumour immune response in splenic lesions.

Histiocytic sarcoma displayed a distinctive immune-related transcriptional profile, with downregulation of *C1QA*, *C1QC*, and *MPEG1*, genes commonly expressed by macrophages and involved in phagocytosis, immune modulation, and macrophage polarization [40,41]. Rather than indicating simple immune suppression, these findings suggest profound reprogramming of the tumour immune microenvironment, raising the question of whether immune dysregulation acts as a driver of histiocytic sarcoma development or emerges as a consequence of malignant histiocytic expansion. Overall, these data support the concept that splenic nodular lesions are characterised not only by cellular proliferation but also by progressive molecular re-education of the stromal, immune, and extracellular matrix compartments. However, this interpretation cannot yet be fully validated by existing veterinary research on the tumour microenvironment of non-angiomatous splenic nodular lesions, as most available studies focus almost exclusively on canine hemangiosarcoma [21]. Consequently, whether similar microenvironmental dynamics operate across other splenic lesion types remains largely unexplored and warrants further investigation.

The main limitation of this study is the small number of samples available for RNA sequencing, which constrained the statistical power for full delineation of carcinogenic pathways. Moreover, while the transcriptomic data suggest potential progression models and functional roles for differentially expressed genes, this study remains observational and cannot establish causality. Functional validation via in vitro or in vivo models will be required to confirm the mechanistic roles of these candidate genes. Finally, due to the scarcity of comparable canine splenic transcriptomic datasets, external validation opportunities are limited at present. In conclusion, this study provides new molecular insights into the pathogenesis of canine splenic nodular lesions by revealing transcriptional changes associated with extracellular matrix remodelling, immune modulation, and iron metabolism. The identification of shared and lesion-specific differentially expressed genes highlights the biological continuum between hyperplastic and neoplastic splenic processes, suggesting that splenic tumorigenesis may evolve through progressive alterations in stromal dynamics and immune regulation. Importantly, our results point towards several potential biomarkers (e.g., SLC40A1, OAS3) whose expression patterns may distinguish benign from malignant lesions and reflect key pathways in tumour progression. In the emerging field of veterinary precision medicine, such biomarkers could eventually improve pre-operative diagnostic accuracy, refine prognostication, or identify novel therapeutic targets (e.g., iron metabolism modulation or stromal ECM inhibitors). Beyond their immediate diagnostic relevance, these results lay the groundwork for future functional and translational investigations aimed at validating molecular drivers of splenic tumorigenesis that would not only improve clinical management of canine splenic disease but also contribute to comparative oncology, enhancing our understanding of shared oncogenic mechanisms across species.

## 5. Conclusions

This study explored gene expression profiles in twelve dogs diagnosed with splenic lymphoid hyperplasia, histiocytic sarcoma, and stromal sarcoma. The analysis revealed shared gene expressions associated with key biological processes, including cell cycle regulation, proliferation, cytoskeletal organization, iron metabolism, and immune response, which contribute to the distinct characteristics of each tumour subtype. The identification of novel genes provides valuable insights into tumour origin and highlights potential diagnostic and therapeutic targets. Overall, the findings underscore the importance of transcriptome analysis as a complement to traditional histological and immunohistochemical methods. Additionally, this study stresses the critical need for rigorous quality control and precise diagnostic techniques, particularly immunohistochemical staining, to prevent misdiagnosis and account for low-quality outlier samples, which could compromise the statistical reliability of biological analyses.

## Figures and Tables

**Table 1:** Immunohistochemical markers for splenic lymphoid hyperplasia, complex hyperplasia, histiocytic sarcoma, and stromal sarcoma.

Antibody	Isotype	Clonality	Manufacturer	Antigen Retrieval	Dilution	Control Tissue
CD3	Rabbit	Polyclonal	Dako (Agilent Technologies Australia, Mulgrave, Victoria, Australia)	HIER, Tris-EDTA, pH 9.0	1:100	Lymph node
CD79a	Mouse	Monoclonal	Dako (Agilent Technologies Australia, Mulgrave, Victoria, Australia)	HIER, Tris-EDTA, pH 9.0	1:300	Lymph node
MAC3817	Mouse	Monoclonal	Dako (Agilent Technologies Australia, Mulgrave, Victoria, Australia)		1:400	Tonsil
IBA-1	Rabbit	Polyclonal	Thermo Fisher Scientific (Scoresby, Victoria, Australia)	Citric Acid, pH 6.0	1:400	Tonsil
Vimentin	Mouse	Monoclonal	Dako (Agilent Technologies Australia, Mulgrave, Victoria, Australia)		1:800	Prostate

**Table 2:** Summary of significantly expressed genes among splenic nodular lesion.

Gene Name	Upregulation or Downregulation	Splenic Lesions	Functional Role	Biomarker Potential
*CSRP1*	Downregulated	All splenic lesions versus normal	Cytoskeletal organisation, cell growth and differentiation	Early cell and tissue dysregulation
*SLC40A1*	Downregulated	All splenic lesions versus normal	Involved in iron transport through ferroportin-1 expressed in macrophages	Target for iron metabolism-based therapies
*MLC1*	Upregulated	SS versus normal	Involved in membrane channel transport, cell-cell interactions, and cell-polarity	Candidate for stromal aggressiveness
*NOTCH3*	Upregulated	SS versus CH	Stromal activation and mesenchymal transition	Suggest stromal remodeling
*COL4A and COL12A1*	Upregulated	CH versus SS	Extracellular matrix remodelling	Potential for invasiveness
*PLOD2*	Upregulated	LH versus SS	Involved in collagen cross-linking and stiffness	Potential for invasiveness and metastasis
*CPXM2 and*	Upregulated	LH versus SS	Proliferation and migration via EMT modulation	Candidate for cancer stemness
*GALNT5*	Upregulated	LH versus SS	Cell functions include signaling, cycle control and survival	Oncogene protein
*TFPI2*	Upregulated	LH versus CH	ECM remodeling, protective by inhibiting proteases	Candidate for early lesion transition
*DLA-12*	Downregulated	LH, SS versus normal	Antigen presentation through MHCI	Reduction of immune surveillance
*FTL*	Downregulated	CH, SS, HS versus normal	Involved in iron metabolism	Target for iron metabolism-based therapies
*OAS3*	Upregulated	CH, SS, HS versus normal	Interferon response immune regulation apoptosis	Candidate for tumour aggressiveness
*C1QA, C1QC*	Downregulated	LH, CH, HS versus normal	Tumour associated macrophages	Reduced immune suppression
*MPEG1*	Downregulated	LH, CH, HS versus normal	Macrophage innate immunity chromosome segregation	Suggest altered macrophage function

## Data Availability

The original contributions presented in this study are included in the article. Further enquiries can be directed to the corresponding author.
